# Treatment of renal angiomyolipoma: pooled analysis of individual patient data

**DOI:** 10.1186/s12894-015-0118-2

**Published:** 2015-12-28

**Authors:** Teele Kuusk, Fausto Biancari, Brian Lane, Conrad Tobert, Steven Campbell, Uri Rimon, Vito D’Andrea, Aare Mehik, Markku H. Vaarala

**Affiliations:** Department of Surgery and Medical Research Center Oulu, Oulu University Hospital and University of Oulu, PO Box 21, 90029 OYS Oulu, Finland; Division of Urology, Michigan State University, Grand Rapids, Michigan USA; Department of Urology, Glickman Urological and Kidney Institute, Cleveland Clinic, Cleveland, Ohio USA; Sheba Medical Center, Tel-Hashomer, Sackler School of Medicine, Tel-Aviv University, Tel-Aviv, Israel

**Keywords:** Angiomyolipoma, Bleeding, Radiofrequency ablation, Surgery, Embolisation, Re-intervention

## Abstract

**Background:**

This study was performed to evaluate the impact of baseline characteristics and treatment methods on the outcome of sporadic renal angiomyolipoma (AML).

**Methods:**

This was a pooled analysis of individual data of 441 patients with AML retrieved from 58 studies and 3 institutional series.

**Results:**

Ninety-three patients underwent nephrectomy, 163 partial nephrectomy/enucleation, 128 embolisation, 19 cryoablation, 6 radiofrequency ablation, and 32 conservative treatment. Their mean follow-up period was 44.5 months. Patients who experienced major bleeding at presentation had significantly larger tumours than did those without bleeding (mean diameter, 10.1 vs. 5.9 cm, respectively; *p <* 0.0001). A total of 9.4 % and 26.4 % of bleeding tumours had a diameter of <4 and <6 cm, respectively. A tumour diameter of ≥8.0 cm (hazard ratio, 2.07; 95 % confidence interval, 1.20–4.77) and the treatment method (*p =* 0.001) were independent predictors of re-intervention. The risk of re-intervention was significantly higher after embolisation, particularly for large tumours (5-year rate of freedom from re-intervention: diameter of ≥8.0 cm, 49.2 %; diameter of <8.0 cm, 74.8 %; *p =* 0.018). Conservatively treated AMLs had a mean baseline diameter of 3.2 ± 2.7 cm; after 41 months, their mean diameter was 3.7 ± 3.1 cm (*p =* 0.109).

**Conclusions:**

The prevalence of major bleeding is high in sporadic AMLs with a diameter of >6 cm. These results suggest that conservative treatment can be considered in AMLs of <6 cm in diameter. Among current treatment methods, embolisation was associated with a significantly higher risk of re-intervention. Further studies are needed to define risk factors for bleeding and assess the relative benefits of different treatment modalities.

## Background

Renal angiomyolipomas (AMLs) are frequent benign renal tumours composed of fat cells, smooth muscle cells, and blood vessels [1–3]. These tumours belong to a family of perivascular epithelioid cell tumours [4]. AMLs occur sporadically in 80 % of cases, whilst the remaining cases are associated with various genetic disorders [2]. The incidence of AMLs in the general population is 0.4 % [5], but this tumour has been reported in 5.7 % to 6.9 % of partially resected, preoperatively presumed cases of renal carcinoma [6, 7]. The most severe complication related to renal AML is retroperitoneal bleeding, which has been reported in 15 % of patients [2] and may lead to shock in 20 % to 30 % of these patients [8, 9].

According to the current guidelines of the European Association of Urology [10], the primary indications for treatment of renal AML are the presence of symptoms or suspected malignancy. Biopsy may guide the treatment decisions for lesions with unusual growth and imaging characteristics [3]. The Level C recommendations for prophylactic intervention include large AMLs, women of childbearing age, and patients for whom follow-up or access to emergency care may be inadequate [10]. The treatment threshold for AML tumours with a diameter of ≥4 cm has recently been disputed. Indeed, a recent study showed that treating all AMLs of >4 cm may lead to an over-treatment rate of 65 % [11]. Additionally, the optimal treatment method for bleeding tumours has not yet been defined [2, 11–14]. The aim of this study was to evaluate the impact of baseline characteristics, particularly tumour diameter, and treatment methods on the outcome of sporadic renal AML.

## Methods

A literature search of PubMed and Scopus was performed in March 2014 using the key words ‘renal’ and ‘angiomyolipoma’. The Preferred Reporting Items for Systematic Reviews and Meta-Analyses (PRISMA) criteria were followed [15, 16]. Adult patients who received any conservative or invasive treatment for renal AML were included in the analysis. Articles reporting on patients with tuberous sclerosis complex or epithelioid AML were excluded. Only articles written in the English language were included in this analysis. Two of the authors individually reviewed the abstracts of the retrieved citations to select relevant series. Data from each series were independently extracted by the two authors and subsequently cross-checked. This literature search identified a number of small studies with heterogeneous treatment strategies and a lack of treatment-specific data on survival and freedom from re-intervention, which prevented the performance of an aggregate survival meta-analysis. Because of these limitations, a pooled analysis of individual patient data was performed. Retrieved articles were reviewed for any data at the individual level that provided information regarding sex, symptoms, indications for invasive or conservative treatment, type of treatment, freedom from re-intervention, and survival. The definition criterion for major bleeding was any sign of retroperitoneal bleeding on imaging examination.

Authors of case series were asked to provide these data in a dedicated Excel spreadsheet. After permission was granted by the Oulu University Hospital’s medical director, data on patients treated at the Oulu University Hospital were retrieved from the electronic records and included in the present analysis. The study was conducted according to the principles of the Helsinki Declaration. For this retrospective chart review, no written informed consent for participation in the study was obtained from participants.

### Statistical analysis

Data were analysed at the individual patient level using SPSS statistical software, version 22.0 (IBM Corp., Armonk, NY, USA). Nominal variables are summarised as counts and percentages, whereas continuous variables are reported as means and standard deviations. Univariate analysis was performed using the Kruskal–Wallis, Mann–Whitney, Wilcoxon, and Fisher exact tests, as appropriate. Freedom from re-intervention and survival were estimated using the Kaplan–Meier method. The impact of different baseline characteristics and operative variables on late outcomes was evaluated using the log-rank test and the Cox proportional hazards method. Tumour size was first included in the multivariate analysis as a continuous variable and then dichotomised according to incremental threshold values from 4.0 to 10.0 cm, respectively. A *p*-value of <0.05 was considered statistically significant.

## Results

**Fig. 1:**
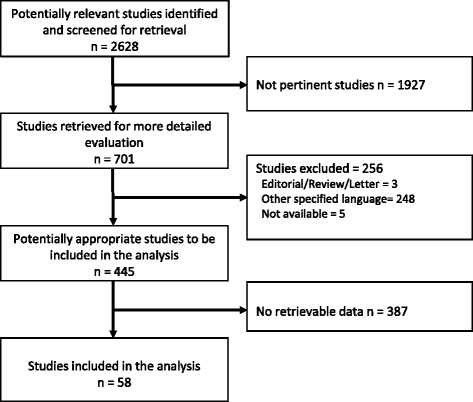
Literature search flow chart

Fifty-eight studies met the inclusion criteria and were suitable for inclusion in the present analysis (Fig. 1). Individual patient data were obtained from the authors of two studies [17, 18] and from the Oulu University Hospital (7 patients). In overall, this dataset included 441 patients with sporadic renal AML who were the subjects of this analysis. Patient characteristics and their outcomes are summarised in Table 1. Ninety-three patients underwent nephrectomy, 163 partial nephrectomy or enucleation, 128 embolisation, 19 cryoablation, 6 radiofrequency ablation, and 32 conservative treatment (Table 1). Their mean follow-up period was 44.5 ± 35.8 months.

**Table 1:** Baseline characteristics and outcomes of patients with renal angiomyolipomas

Variables	Overall	Conservative treatment	Cryoablation	Embolisation	Radiofrequency ablation	Partial nephrectomy/ enucleation	Nephrectomy	*P*-value
No. of patients	441	32	19	128	6	163	93	
Age (years)	51.5 ± 14.5	53.2 ± 15.0	53.6 ± 14.8	47.9 ± 14.7	60.4 ± 6.9	52.9 ± 13.7	52.4 ± 14.9	0.020
Females	353 (80.4)	24 (75.0)	18 (94.7)	103 (80.5)	5 (80)	129 (79.6)	74 (79.6)	0.494
Imaging method								<0.0001
US	9 (9.4)	3 (9.4)	0	2 (1.6)	0	2 (1.2)	2 (2.2)	
CT	411 (93.6)	26 (81.3)	8 (42.1)	125 (97.7)	6 (100)	160 (98.2)	86 (94.5)	
MRI	8 (1.8)	3 (9.4)	0	1 (0.8)	0	1 (0.6)	3 (3.3)	
CT/MRI	11 (2.5)	0	11 (57.9)	0	0	0	0	
Tumour diameter (cm)	6.5 ± 5.0	4.4 ± 5.1	2.6 ± 1.6	9.1 ± 4.8	3.9 ± 2.5	4.7 ± 4.1	7.6 ± 6.0	<0.0001
Bleeding	54 (12.2)	4 (12.5)	0	32 (25.0)	0	7 (4.3)	10 (10.8)	<0.0001
3-year survival	97.9 %	100 %	94.7 %	100 %	100 %	95.6 %	98.4 %	0.037
Reintervention	41 (9.4)	1 (3.1)	0	38 (29.7)	0	2 (1.2)	0	<0.0001
Surgery	18 (4.1)	0	0	17 (13.3)	0	0	0	<0.0001
3-year freedom from reintervention	87.8 %	96.9 %	100 %	63.5 %	100 %	98.2 %	100 %	<0.0001

Data were obtained from the overall series, based on treatment strategy. Nominal variables are reported as counts and proportions; continuous variable are reported as mean and standard deviation

**Fig. 2:**
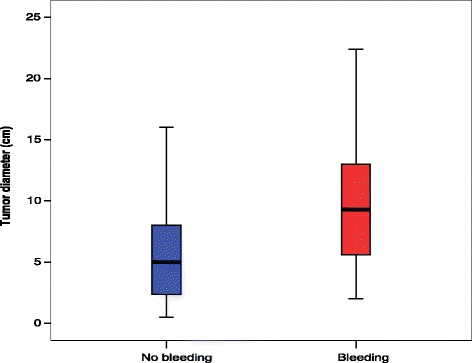
Box plot showing impact of baseline tumour size on severe bleeding. Fifty-four of 441 patients had severe bleeding at presentation (*p <* 0.0001)

Patients presenting with major retroperitoneal bleeding (54 of 441 patients) had significantly larger tumours than did patients without bleeding (mean maximal tumour diameter, 10.1 ± 5.9 vs. 5.9 ± 4.7 cm, respectively; *p <* 0.0001) (Fig. 2). Among the bleeding tumours, 5 of 54 (9.4 %) and 14 of 54 (26.4 %) had maximal diameters of <4 and <6 cm, respectively.

**Fig. 3:**
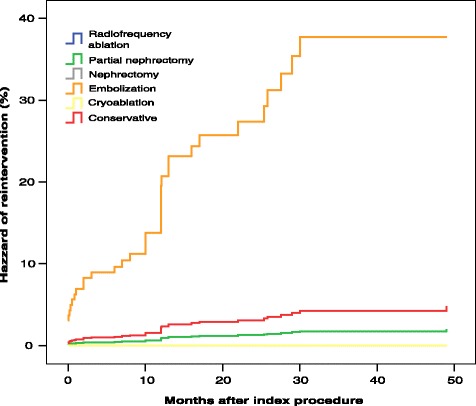
Cox proportional hazards model for repeat intervention. The model was created according to different treatment strategies and adjusted for tumour diameter, presence of bleeding, age, and sex. The freedom from re-intervention curves after radiofrequency ablation and nephrectomy are behind the cryoablation curve in the figure

A Cox proportional hazards model including sex (*p =* 0.29), age (*p =* 0.38), tumour size (*p =* 0.24), presence of major bleeding (*p =* 0.86), and treatment modality (*p =* 0.003) showed that the treatment method was the only independent predictor of re-intervention. When the baseline tumour diameter was dichotomised with an 8.0-cm cutoff, the regression analysis showed that the treatment modality (*p =* 0.001) (Fig. 3) and a baseline tumour diameter of ≥8.0 cm (*p =* 0.013; hazard ratio [HR], 2.07; 95 % confidence interval [95 % CI], 1.20–4.77) were independent predictors of re-intervention. The risk of re-intervention was particularly evident in patients who had undergone embolisation (Fig. 3). Because of this, further analyses were performed only in the subset of patients who underwent embolisation treatment.

Among 128 patients who underwent embolisation, a Cox proportional hazards model including age, sex, baseline tumour diameter, major bleeding, and tumour diameter showed that only a tumour diameter of ≥8.0 cm was an independent predictor of re-intervention (*p =* 0.017; HR, 2.36; 95 % CI, 1.17–4.79). The 5-year actuarial estimate of freedom from re-intervention after embolisation in patients with a tumour diameter of ≥8.0 cm was 49.2 %, whereas it was 74.8 % for patients with smaller tumours (log-rank test, *p =* 0.013).

Among the 32 patients who were treated conservatively, the mean initial diameter was 3.2 ± 2.7 cm (range, 1.5–14.0 cm), whereas it was 3.7 ± 3.1 cm (range, 1.5–14.0 cm) at the end of the mean follow-up period (41 ± 38 months) (Wilcoxon test, *p =* 0.109). Only 3 of these patients presenting with tumour diameters of 2.5, 4.0, and 7.0 cm demonstrated tumour growth to 6.5, 7.8, and 9.0 cm, respectively. The latter tumours were still treated conservatively at the last follow-up.

## Discussion

The treatment strategy for renal AMLs is based mainly on evidence acquired during the 1980s [8]. Because major bleeding is the most severe complication of AML, prophylactic treatment may be indicated to avoid this haemorrhagic event. For many years, the threshold diameter for prophylactic treatment has been 4 cm [8]; however, this threshold has recently been disputed [11, 19]. Indeed, the diagnostic methods for AMLs have improved significantly during recent years, and the indication and efficacy of invasive treatment strategies for AML should be re-evaluated in light of recent studies. We performed a literature review covering the era of modern diagnostic modalities to obtain detailed patient data on the efficacy of the current treatment strategies. Most of the published data were heterogeneous and presented significant biases. Therefore, we proceeded with a pooled analysis of all individual patient data available in the literature and included data from two patient series [17, 18] along with patient data from the Oulu University Hospital, Finland.

The present analysis showed that the risk of bleeding is associated with a large tumour diameter. Although even small AMLs are known to bleed, only 10 % of bleeding tumours in our analysis were below the traditional prophylactic treatment threshold of 4 cm. In fact, among bleeding tumours, 5 of 54 (9.4 %) and 14 of 54 (26.4 %) were <4 and <6 cm in diameter, respectively. This suggests that a diameter cutoff for treatment could be appropriately set at >6 cm. Indeed, a recent study suggested that only 34 % of patients with tumours of ≥4 cm required intervention [11]. Moreover, 67 % of symptomatic patients were managed with active surveillance and without late intervention [11]. Minimal or no growth of sporadic AMLs was similarly observed in earlier studies [8, 19, 20].

The prediction of severe bleeding events associated with AML is an important clinical issue and may dictate the prophylactic treatment strategy. A number of studies have suggested that the risk of bleeding is related to the vascularity and size of the tumour [21–23]. Rimon et al. [21] concluded that large (>4 cm) AMLs with minimal vascularity are less likely to bleed and that a grading system, based on tumour vascularity determined using digital subtraction angiography, may help to select patients needing embolisation. AMLs with a dominant large feeding vessel may be optimal for embolisation.

Although several studies [12, 24–26] have shown excellent outcomes with different treatment modalities, the present results suggest suboptimal outcomes after AML embolisation. In the past, embolisation was the treatment of choice for AMLs [19] probably because analyses of different treatment modalities have shown that embolisation is effective for bleeding tumours [19]. Preservation of the renal parenchyma [27, 28], effective occlusion of bleeding vessels [26], and surgery prevention [2] are considered the main benefits of embolisation. However, in our study, the 3-year rate of freedom from re-intervention after embolisation was 63.5 %, whereas it approached 100 % for all other treatment modalities. Embolisation was performed in 128 tumours, 30.5 % of which were asymptomatic and 25.0 % of which were bleeding. Tumours treated by embolisation also had the largest mean diameter (9.1 ± 4.8 cm). Remarkably, only the treatment modality was associated with a risk of re-intervention, consistent with the results of other series [12, 19, 24–26, 28]. We observed that the risk of re-intervention was lower if embolisation was performed for bleeding tumours with a diameter of <8 cm. Embolisation is less effective than partial nephrectomy for tumours of >8 cm likely because of their high vascularity, making embolisation of these large tumours more complex and less efficacious.

Although the number of patients who underwent invasive methods in our included studies was rather small, no re-interventions after either cryoablation or radiofrequency ablation were reported (Table 1). Castle et al. [29] reported no recurrence after radiofrequency ablation of AMLs with a mean diameter of 2.6 cm. It has been suggested that radiofrequency ablation may also be a valid treatment option for larger tumours [30]. However, post-treatment retroperitoneal bleeding due to fracture of the tumour mass during the procedure may be a significant concern if larger AMLs are treated with cryoablation [31, 32].

The data presented herein support the current strategy for surveillance in asymptomatic patients. Based on our results and those presented earlier [11], aggressive use of prophylactic treatment should be avoided to prevent over-treatment. Based on the present data and a prior study [33], surgery provides good results for larger bleeding AMLs. A 3.4 % recurrence rate among surgically treated patients was observed during a median follow-up of 8 years [33]. However, the risk of major postsurgical complications (range, 7 % to 12 %) [33, 34] is far higher than that of severe adverse events after embolisation [12]. Elective surgical treatment after emergency embolisation performed to stop bleeding may be the treatment of choice for patients not at high risk for major surgery. Additional data are needed to assess the efficacy and durability of radiofrequency ablation and cryoablation in this setting. Prospective studies are warranted to better evaluate the natural course of renal AML, assess the risk factors for bleeding, and compare the different treatment modalities. AMLs that tend to bleed, even small AMLs, may have different radiological and vasculature characteristics [35]. Prospective patient series are needed to reliably evaluate these aspects.

The results of this study may be affected by a number of limitations that should be acknowledged. All studies included in this study were of a retrospective nature with limited follow-up. Data on major bleeding were from non-consecutive, non-longitudinal studies, which might have introduced a bias in the analysis of the prevalence of this complication. Because embolisation has been widely used as a prophylactic treatment of AMLs since the 1990s, publications may be biased toward reporting of complications and alternative surgical treatment modalities.

## Conclusions

The present analysis showed that the prevalence of major bleeding is significantly higher in AMLs larger than 6 cm. Therefore, conservative treatment can be considered for AMLs less than 6 cm in diameter, whereas a threshold for invasive treatment of 4 cm may not be appropriate. Among treatment methods, embolisation was associated with a significantly higher risk of re-intervention. Further studies are needed to define risk factors for bleeding and assess the relative benefits of different treatment modalities.

## Abbreviations

AML: angiomyolipoma
HR: hazard ratio
CI: confidence interval
